# Supplementary material for: Insulin Receptor-Related Receptor Regulates the Rate of Early Development in Xenopus laevis
Source: Int J Mol Sci. 2022 Aug 17;23(16):9250. doi: 10.3390/ijms23169250 (PMC9409083; doi:10.3390/ijms23169250)
Supplement: Supplementary file 1 [file ijms-23-09250-s001.zip › description of supplement figures, files and table.pdf]

Supplement Figure S1. Testing of *insrr* MO2.

A. Embryos injected with *insrr* MO2 at pH 7.2 and 8.5.

B. Embryos injected with *control* MO at pH 7.2.

C. Embryos injected with control MO at pH 8.5.

The diagrams on A', B', and C' demonstrate the percentage distribution of embryos, some of which are shown on A, B, and C, by stage of development.

Supplement Figure S2. Rescue of *insrr* MO1 effect by the co-injected *insrr* mRNA.

A-C. Embryos injected with *control* MO (A), *insrr* MO1 (B), and the mixture of *insrr* MO1 and *insrr* mRNA (C) at pH 7.2.

The diagrams on A', B', and C' demonstrate the percentage distribution of embryos, some of which are shown on A, B, and C, by stage of development.

Supplement Figure S3. Analysis of MO specificity using myc-tagged fragments of xIRR cDNA. The obtained cDNA fragments were sub-cloned into pCS2-twsg1-myc plasmid instead of twsg1 coding frame. To obtain capped mRNA, these plasmids were linearized and mRNAs were synthesized using mMESSAGEmMACHINE kit. For injection experiments MOs were diluted to the final concentration 0.3 mM; mRNAs were diluted to the final concentration 25ng/µl. For injections, mRNA or MO solutions were mixed with FLD (Fluorescein Lysinated Dextran, 40 kDa, 5 mg/mL, Invitrogen) and 4–5 nL of the mixture were injected into single blastomeres at two-cell stage. Injected embryos were cultivated until stage 12, then lysed and expression was analyzed by Western blotting. Coomassie stained gels were used as loading controls.

Supplement Figure S4. A. Annotated list of DEGs (padj<0.05) between *insrr* MO and control MO injected embryos at pH 7.2. **B.** Annotated list of DEGs (padj<0.05) between *insrr* MO injected embryos at pH 8.5 and pH 7.2.

Supplement Figure S5. **A.** The intersection of DEGs (929 DEGs, padj<0.01) between transcriptomes of *insrr* MO and control MO injected embryos at pH 7.2, and DEGs (634 DEGs, padj<0.01) between transcriptomes of *insrr* MO injected embryos at pH 8.5 and pH 7.2. **B**. Analysis of the fold changes of DEGs intersection between trancriptomes of *insrr* MO and control MO injected embryos at pH 7.2 (red color) and between transcriptomes of *insrr* MO injected embryos at pH 8.5 and pH 7.2 (blue color). X axis indicates number of gene and genes were sorted by increasing of the FoldChange between transcriptomes of *insrr* MO injected embryos at pH 8.5 and pH 7.2. **C.** Annotated list of 331 DEGs intersection.

Supplement Figure S6. A. Plot of changing the expression modulus DEGs (929 DEGs, padj<0.01) between transcriptomes of *insrr* MO and control MO injected embryos at pH 7.2. Intersection DEGs (331 genes) indicated by blue color and other genes by orange color. **B.** Plot of changing the expression modulus DEGs (634 DEGs, padj<0.01) between transcriptomes of *insrr* MO injected embryos at pH 8.5 and pH 7.2. Intersection DEGs (331 genes) indicated by blue color and other genes by orange color

Supplementary Table S1. Temporal expression profiles of 30 selected DEGs from Xenbase online database (https://www.xenbase.org)

Supplement file S1. RNA-seq data of all four groups of *Xenopus laevis* embryos (control pH 7.2, control pH 8.5, *insrr* MO 7.2 and *insrr* MO 8.5)

Supplement file S2. Analysis of gene expression between *Xenopus laevis* embryos at stage10 and stage15 using raw data from [19]

Supplement file S3. List of 331 DEGs**,** that were obtained as the intersection of 929 *insrr* MO 7.2/control MO 7.2 DEGs (padj<0.01) and 634 *insrr* MO 8.5/*insrr* MO 7.2 DEGs (padj<0.01).

Supplement file S4. Gene Ontology analysis of upregulated genes between control MO pH 7.2 and control MO 8.5 groups.

Supplement file S5. Gene Ontology analysis of downregulated genes between control MO pH 7.2 and control MO 8.5 groups.
