# Supplementary material for: Insulin Receptor-Related Receptor Regulates the Rate of Early Development in Xenopus laevis
Source: Int J Mol Sci. 2022 Aug 17;23(16):9250. doi: 10.3390/ijms23169250 (PMC9409083; doi:10.3390/ijms23169250)
Supplement: Supplementary file 1 [file ijms-23-09250-s001.zip › Supplement Figures and Table.pdf]

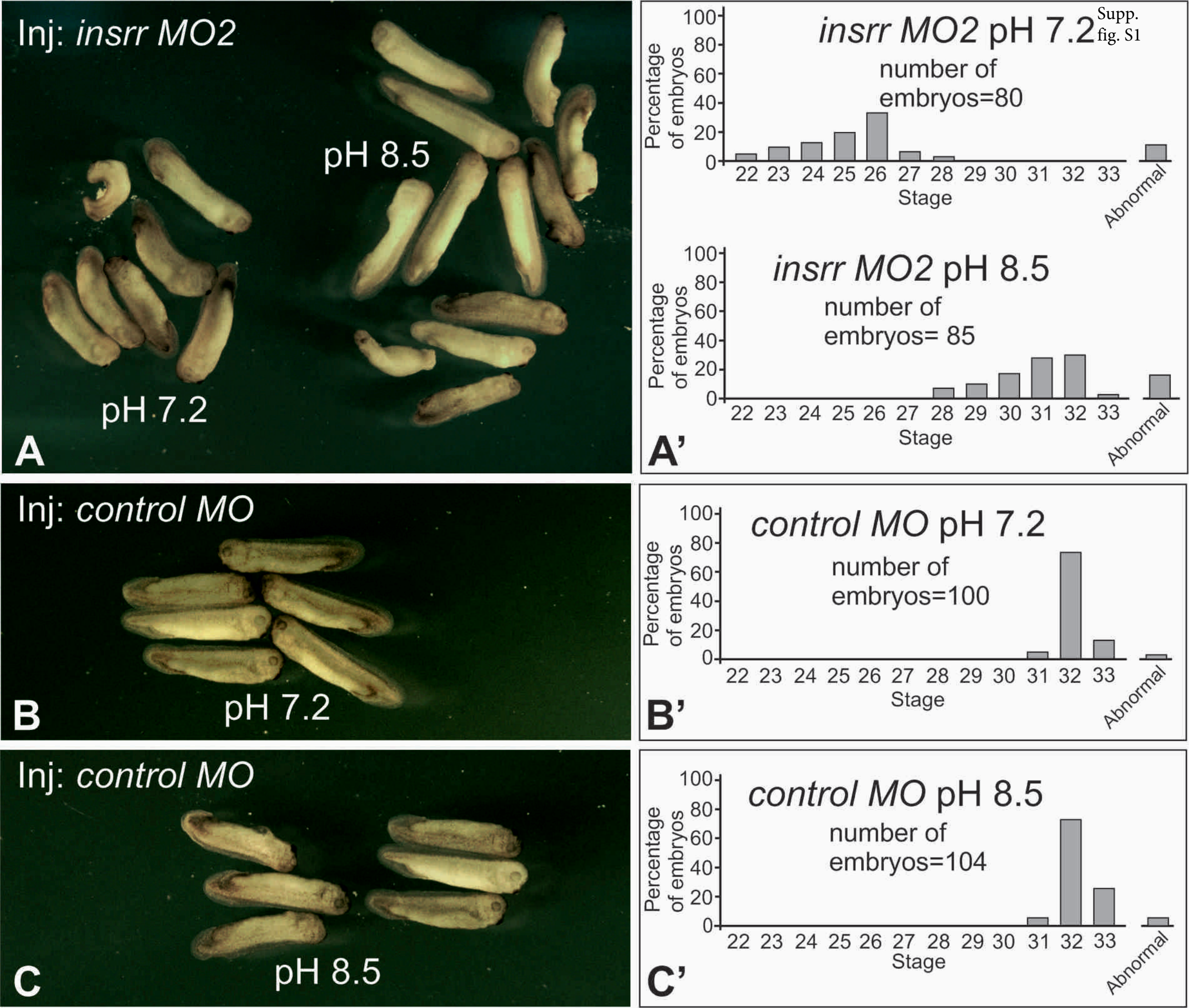

Supp. fig. S1
Inj: insrr MO2
pH 8.5
pH 7.2
A
insrr MO2 pH 7.2
number of embryos=80
Percentage of embryos
Stage
Abnormal
insrr MO2 pH 8.5
number of embryos= 85
A'
Inj: control MO
pH 7.2
B
control MO pH 7.2
number of embryos=100
B'
Inj: control MO
pH 8.5
C
control MO pH 8.5
number of embryos=104
C'

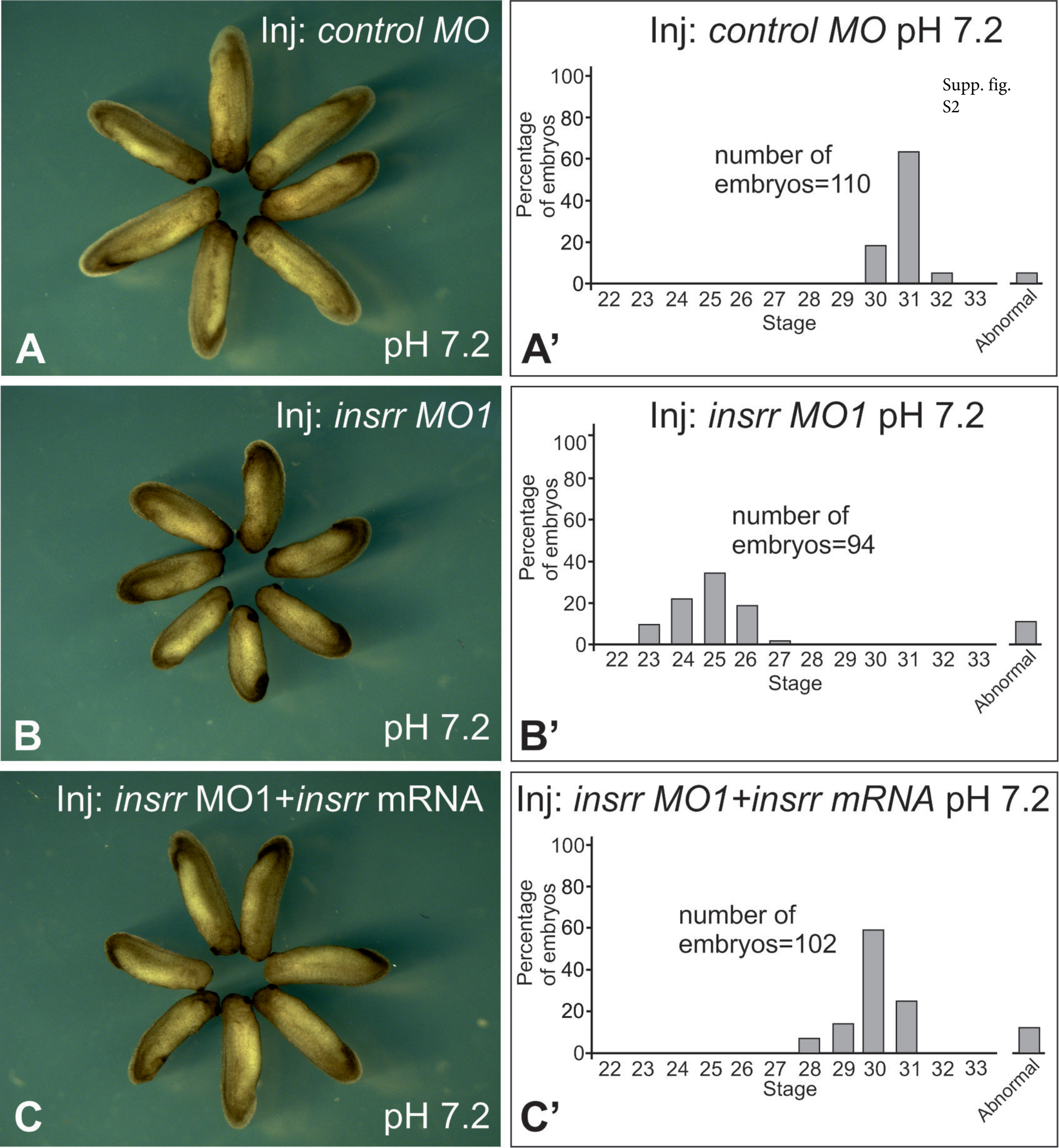

Inj: control MO
pH 7.2
A
Inj: control MO pH 7.2
Supp. fig. S2
number of embryos=110
Percentage of embryos
Stage
22 23 24 25 26 27 28 29 30 31 32 33 Abnormal
A’
Inj: insrr MO1
pH 7.2
B
Inj: insrr MO1 pH 7.2
number of embryos=94
Percentage of embryos
Stage
22 23 24 25 26 27 28 29 30 31 32 33 Abnormal
B’
Inj: insrr MO1+insrr mRNA
pH 7.2
C
Inj: insrr MO1+insrr mRNA pH 7.2
number of embryos=102
Percentage of embryos
Stage
22 23 24 25 26 27 28 29 30 31 32 33 Abnormal
C’

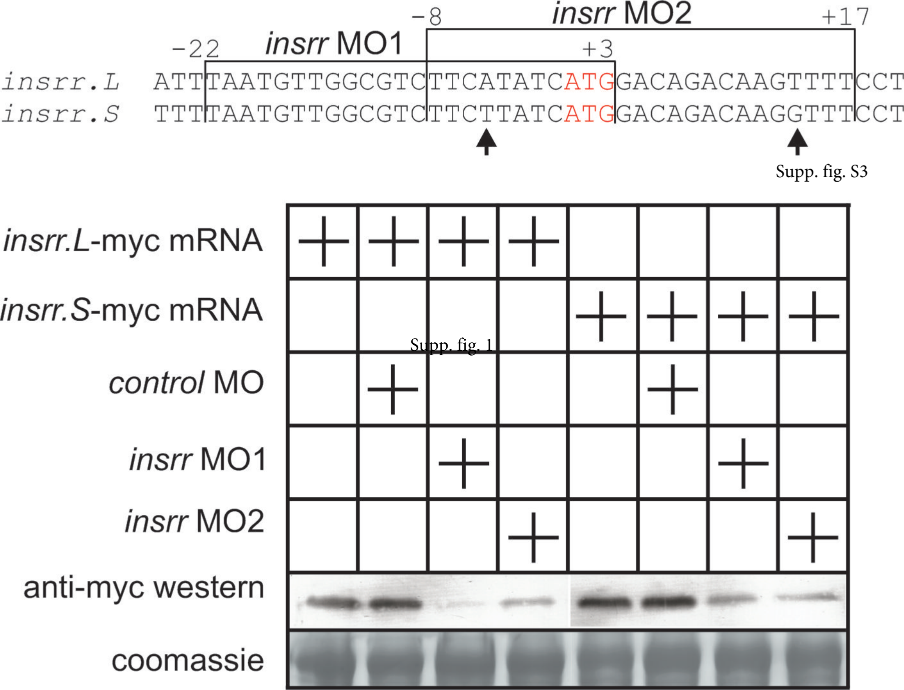

insrr MO2
-8
+17
-22
insrr MO1
+3
insrr.L ATTTAATGTTGGCGTCTTCATATCATGGACAGACAAGTTTTCCT
insrr.S TTTTAATGTTGGCGTCTTCTTATCATGGACAGACAAGGTTTCCT
Supp. fig. S3
insrr.L-myc mRNA
insrr.S-myc mRNA
Supp. fig. 1
control MO
insrr MO1
insrr MO2
anti-myc western
coomassie

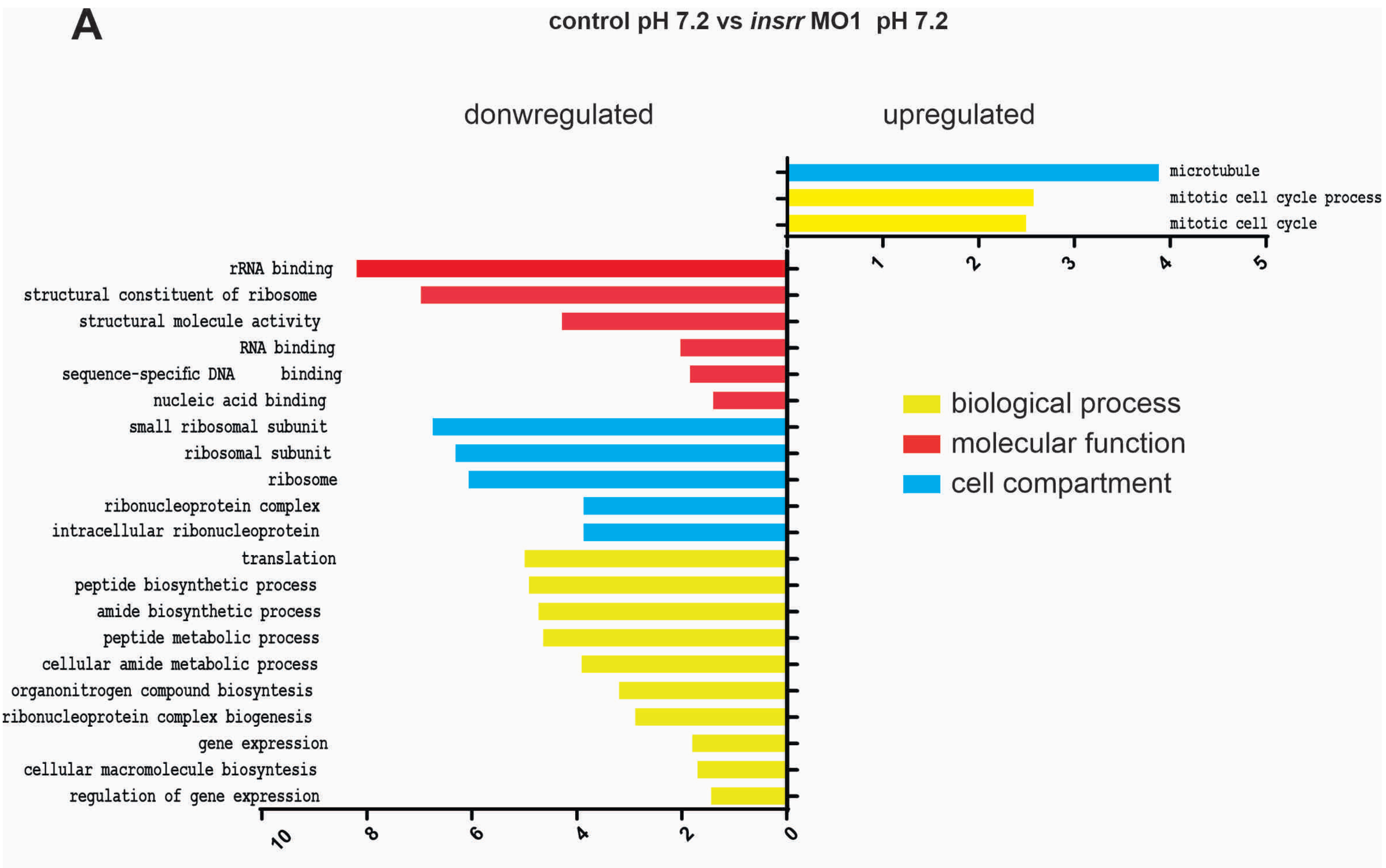
A
control pH 7.2 vs insrr MO1 pH 7.2
donwregulated
upregulated
microtubule
mitotic cell cycle process
mitotic cell cycle
rRNA binding
structural constituent of ribosome
structural molecule activity
RNA binding
sequence-specific DNA binding
nucleic acid binding
small ribosomal subunit
ribosomal subunit
ribosome
ribonucleoprotein complex
intracellular ribonucleoprotein
translation
peptide biosynthetic process
amide biosynthetic process
peptide metabolic process
cellular amide metabolic process
organonitrogen compound biosyntesis
ribonucleoprotein complex biogenesis
gene expression
cellular macromolecule biosyntesis
regulation of gene expression
biological process
molecular function
cell compartment
1 2 3 4 5
10 8 6 4 2 0

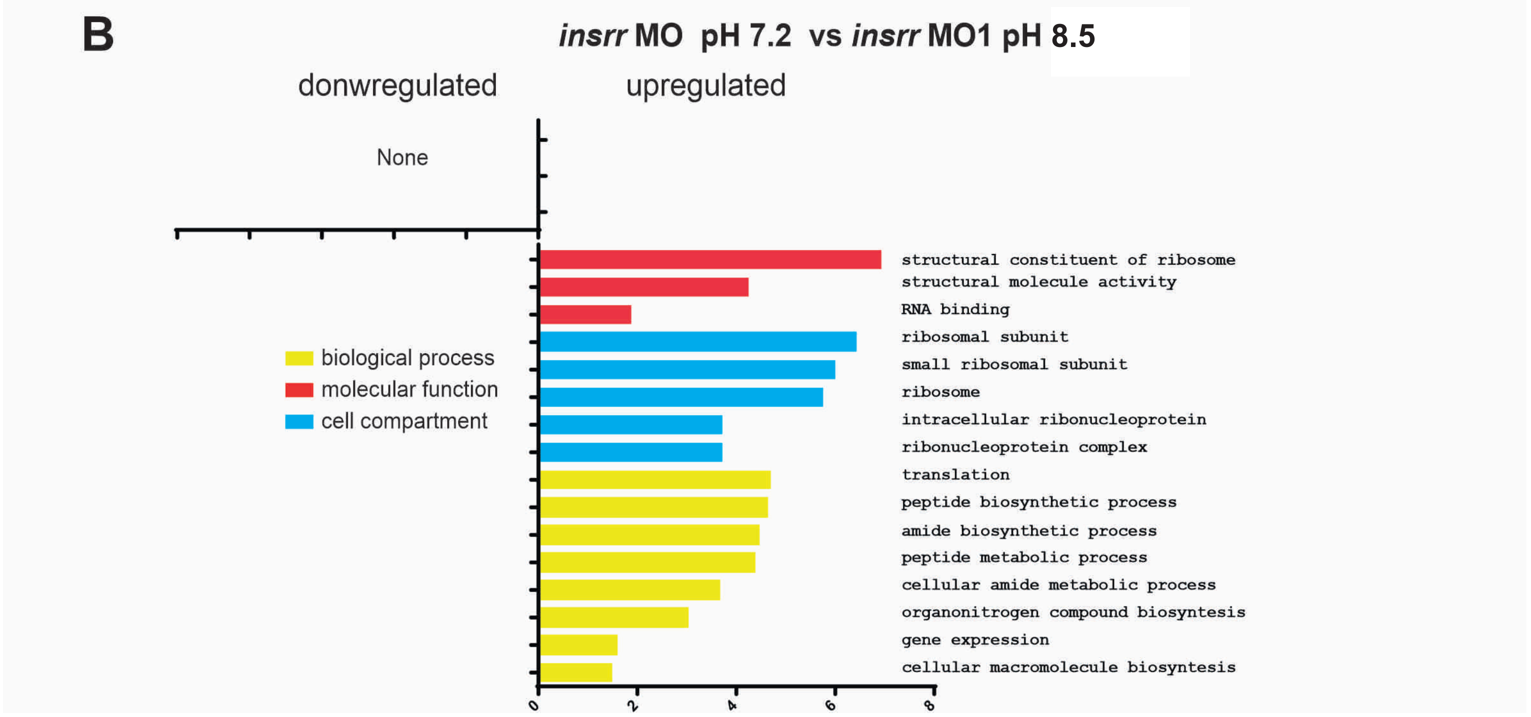
B
insrr MO pH 7.2 vs insrr MO1 pH 8.5
donwregulated
upregulated
None
structural constituent of ribosome
structural molecule activity
RNA binding
ribosomal subunit
small ribosomal subunit
ribosome
intracellular ribonucleoprotein
ribonucleoprotein complex
translation
peptide biosynthetic process
amide biosynthetic process
peptide metabolic process
cellular amide metabolic process
organonitrogen compound biosyntesis
gene expression
cellular macromolecule biosyntesis
biological process
molecular function
cell compartment
0 2 4 6 8

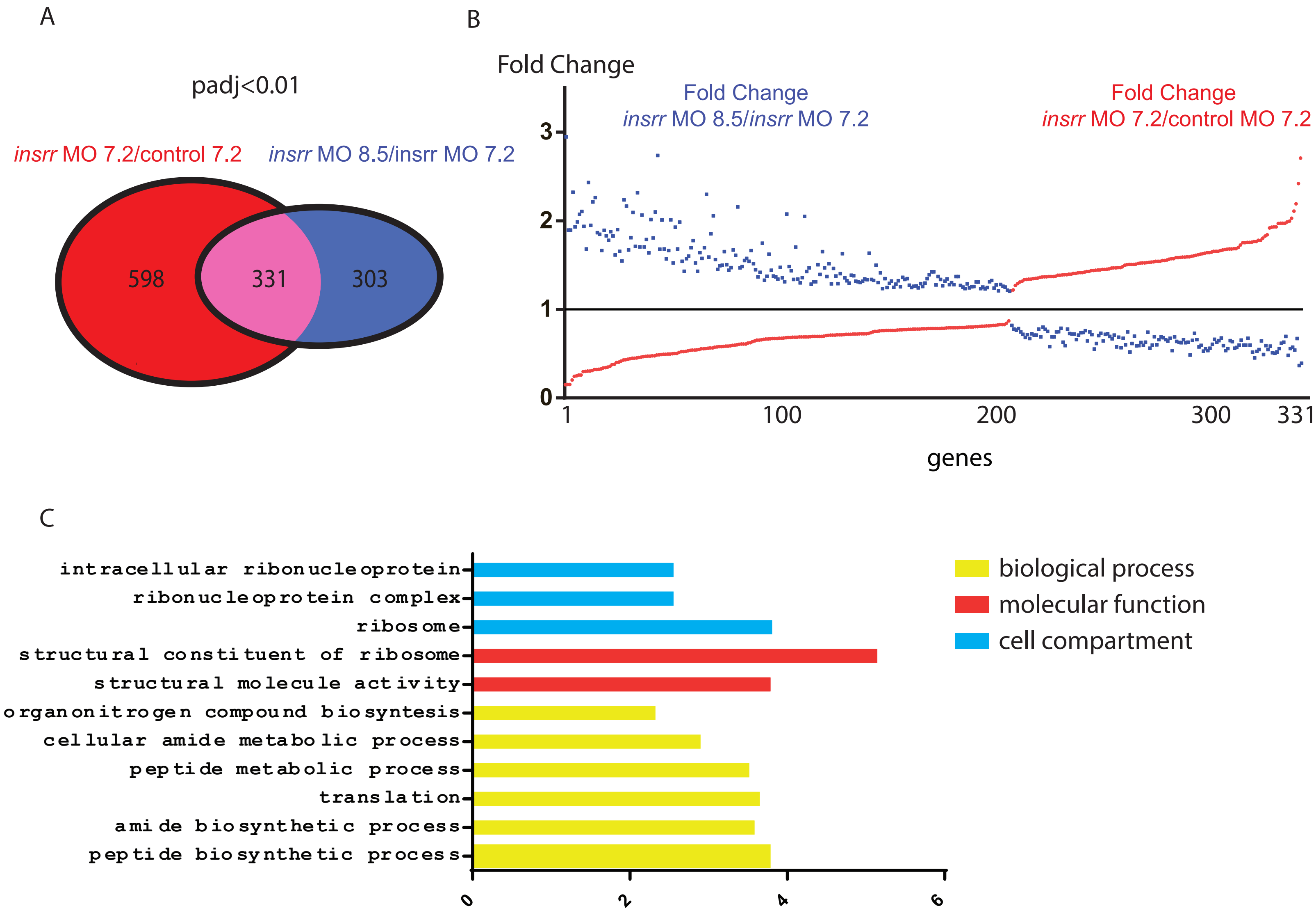
A
padj<0.01
insrr MO 7.2/control 7.2
insrr MO 8.5/insrr MO 7.2
598
331
303
B
Fold Change
Fold Change
insrr MO 8.5/insrr MO 7.2
Fold Change
insrr MO 7.2/control MO 7.2
3
2
1
0
1
100
200
300
331
genes
C
intracellular ribonucleoprotein
ribonucleoprotein complex
ribosome
structural constituent of ribosome
structural molecule activity
organonitrogen compound biosyntesis
cellular amide metabolic process
peptide metabolic process
translation
amide biosynthetic process
peptide biosynthetic process
0
2
4
6
biological process
molecular function
cell compartment

A

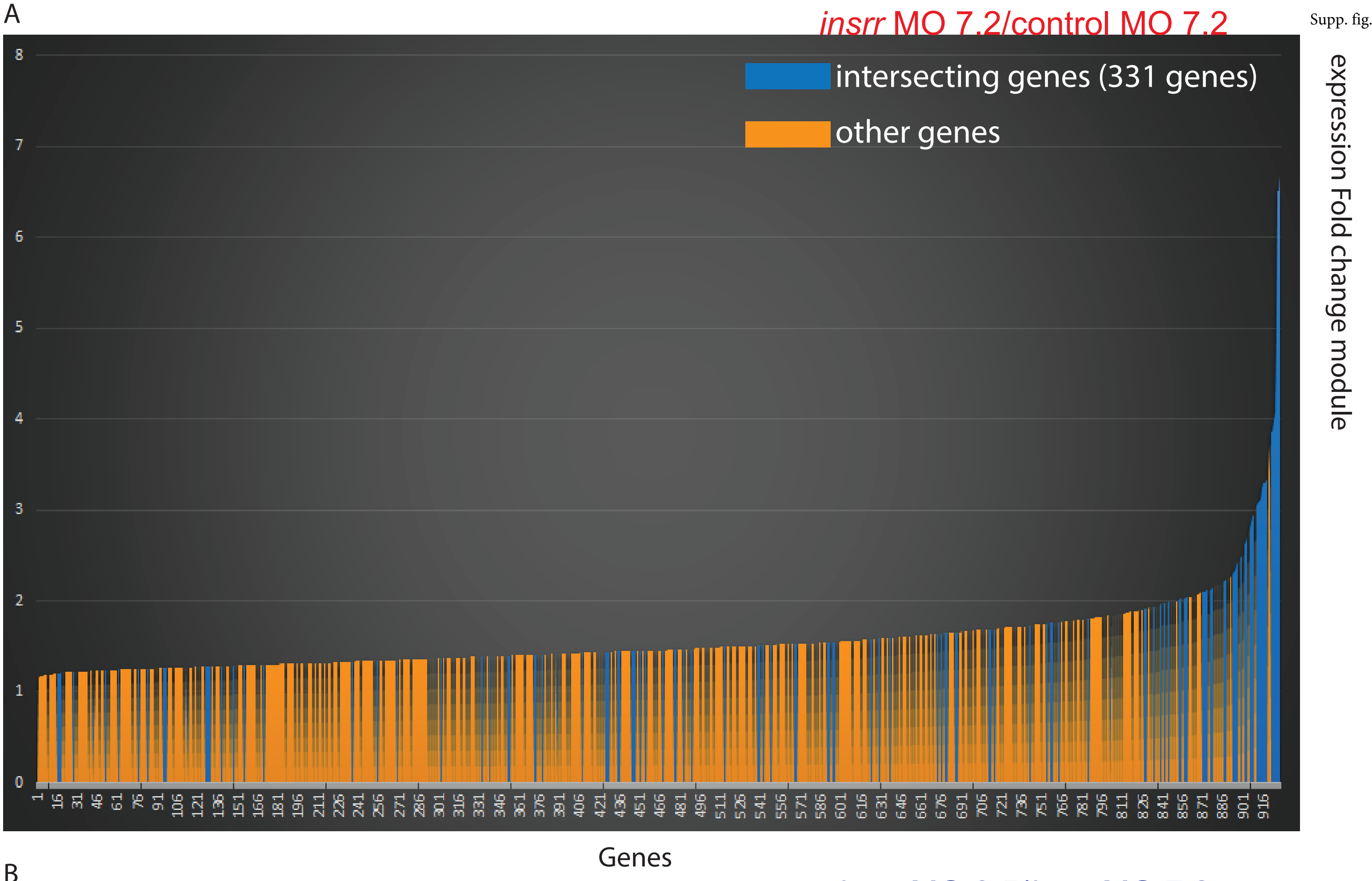
insrr MO 7.2/control MO 7.2
intersecting genes (331 genes)
other genes
expression Fold change module
Genes

B

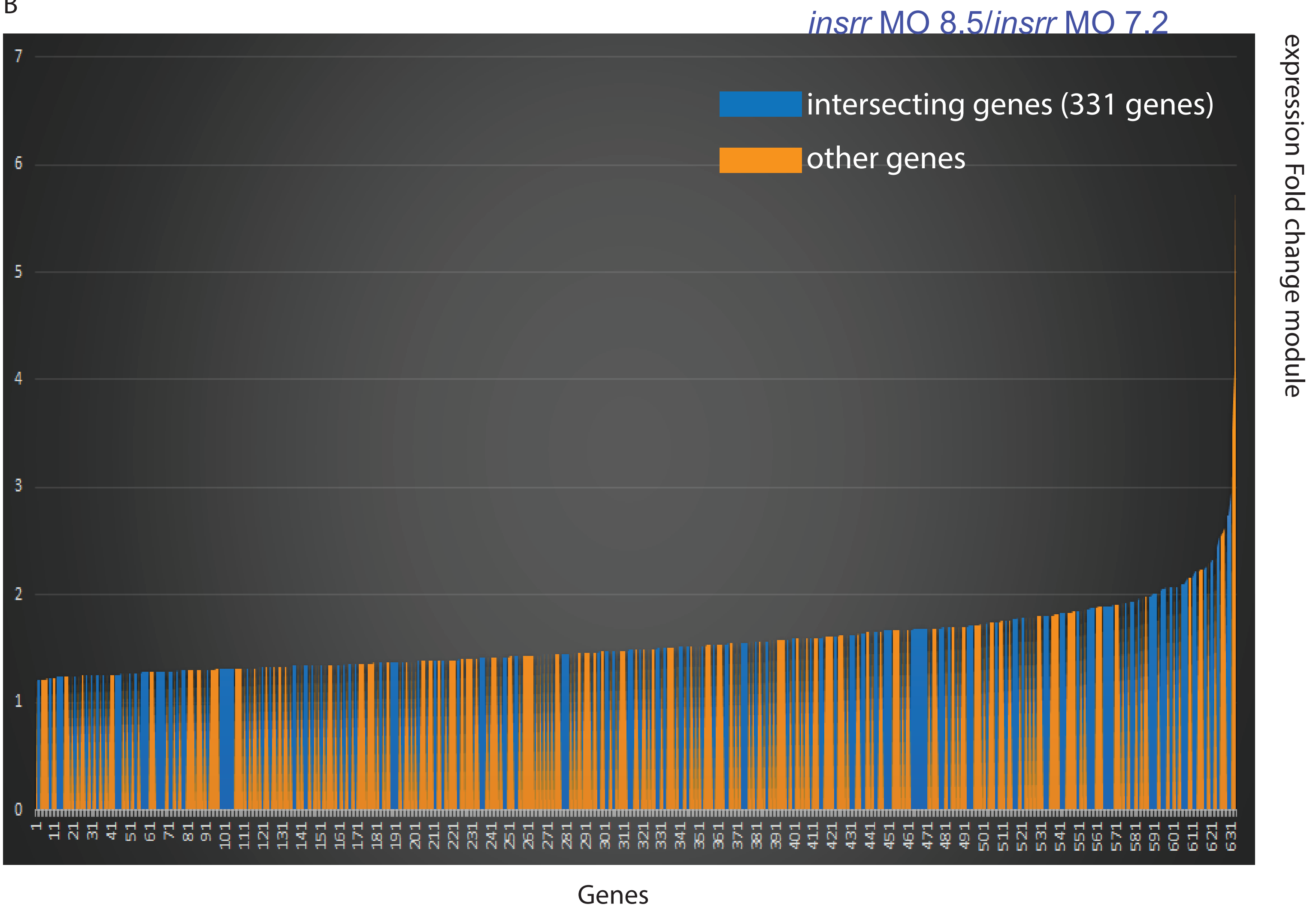
insrr MO 8.5/*insrr* MO 7.2
intersecting genes (331 genes)
other genes
expression Fold change module
Genes

Supplementary table S1. Temporal expression profiles of 30 selected DEGs from Xenbase online database (https://www.xenbase.org)

**Genes upregulated in embryos injected with *insrr* MO1 at the middle neurula stage (marked by vertical red line)**		
Gene	Expression fold change: *insrr* MO1/ *control* MO injected	Temporal expression pattern
larp6-like.1.S	3,70	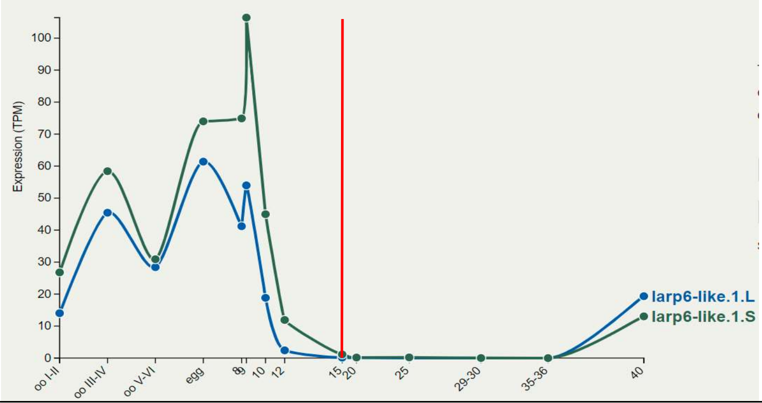
fitm2.L	2,78	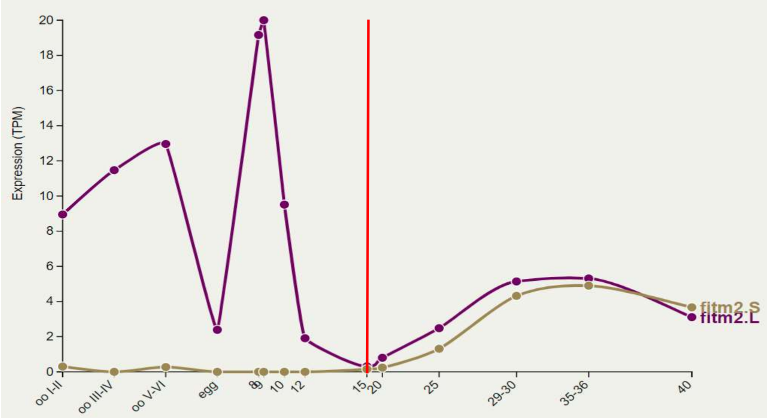
frzb.S	2,70	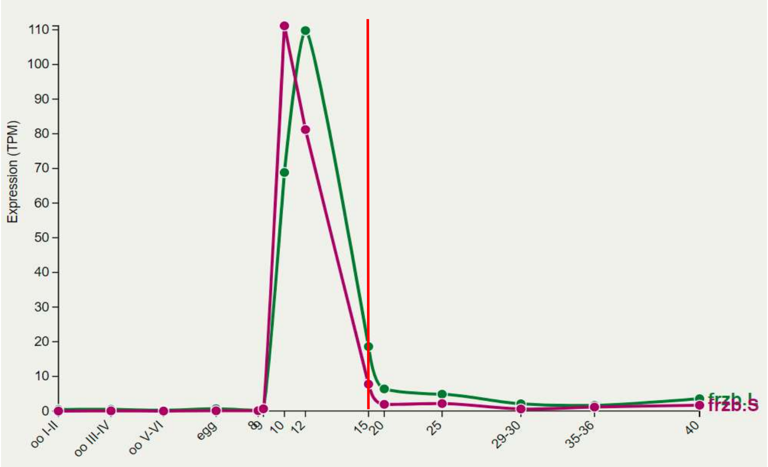

zpd.L	2,50	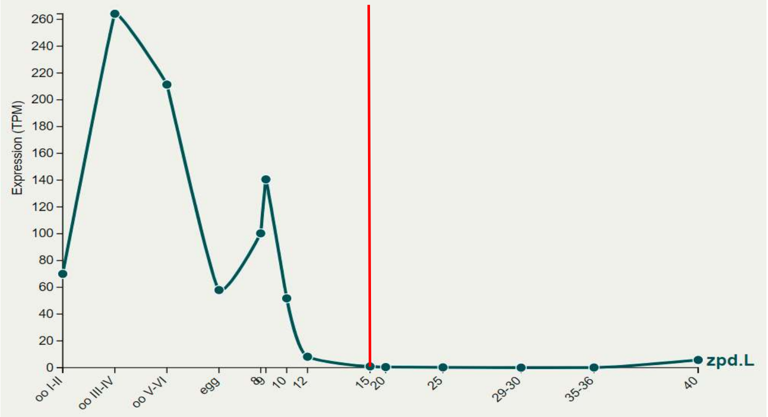
eomes.S	2,44	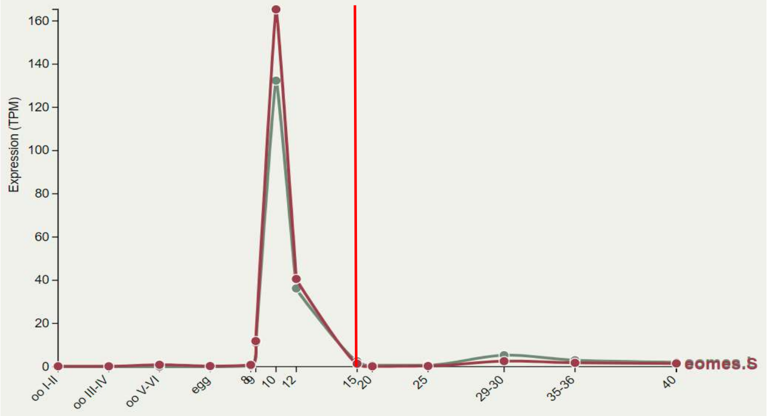
tapt1.S	2,27	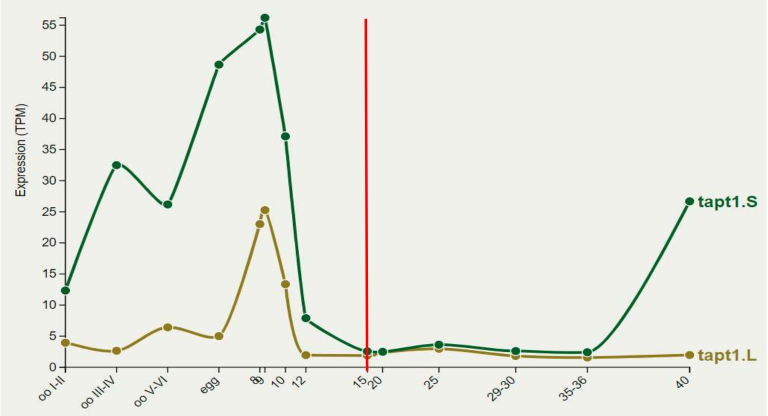
xpo6.S	2,17	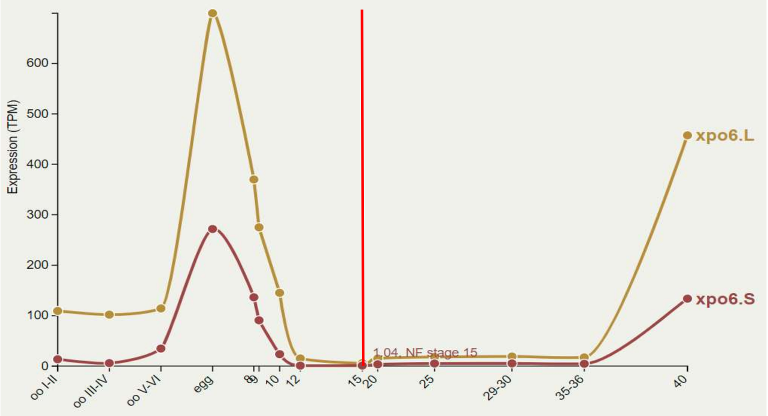

<table>
<tr><td>ccna2.L</td><td>2,17</td><td>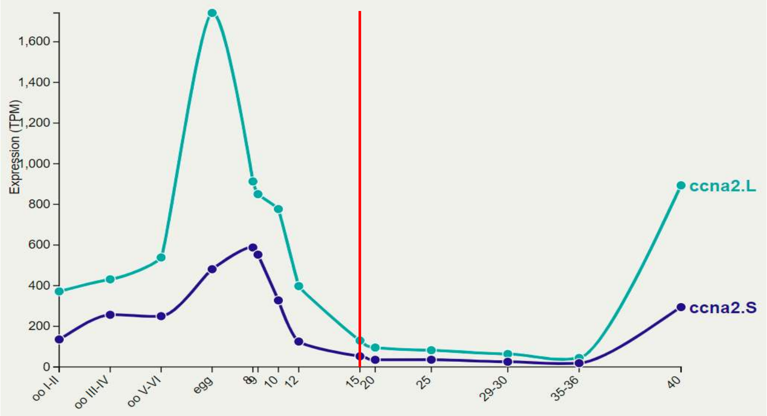
</td></tr>
<tr><td>patl2.L</td><td>2,17</td><td>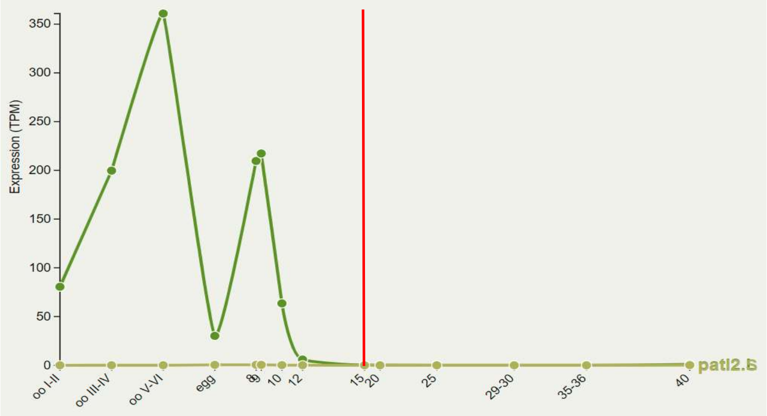
</td></tr>
<tr><td>cer1.S</td><td>2,13</td><td>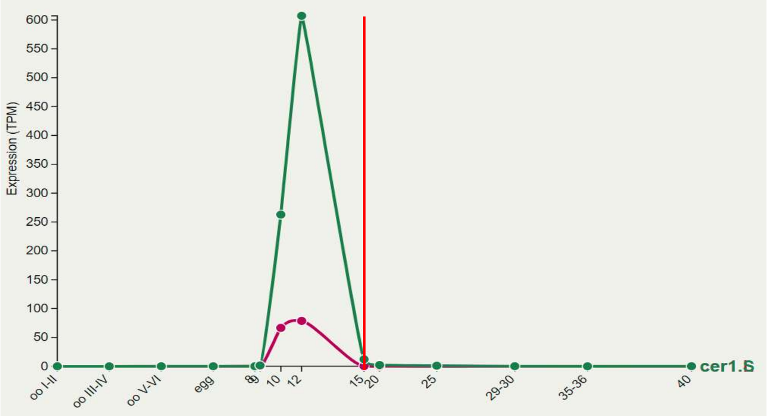
</td></tr>
<tr><td>mapre3.S</td><td>2,04</td><td>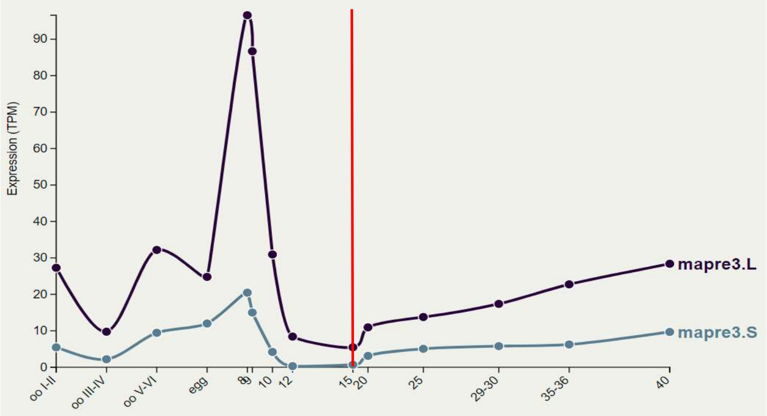
</td></tr>
</table>

cdk5r2.S	2,04	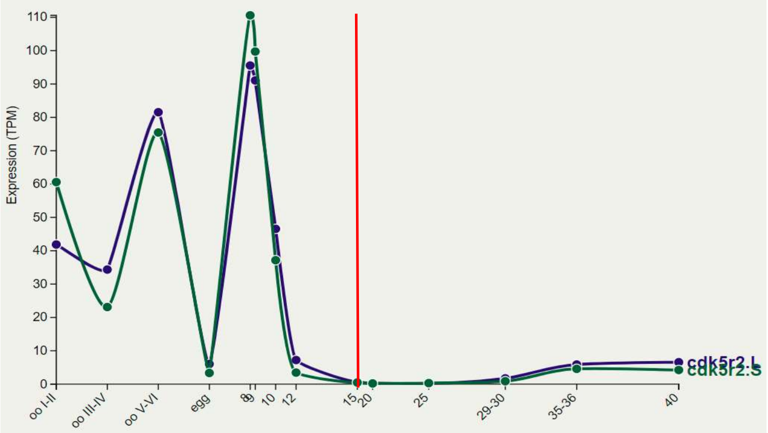
neu1.L	2,00	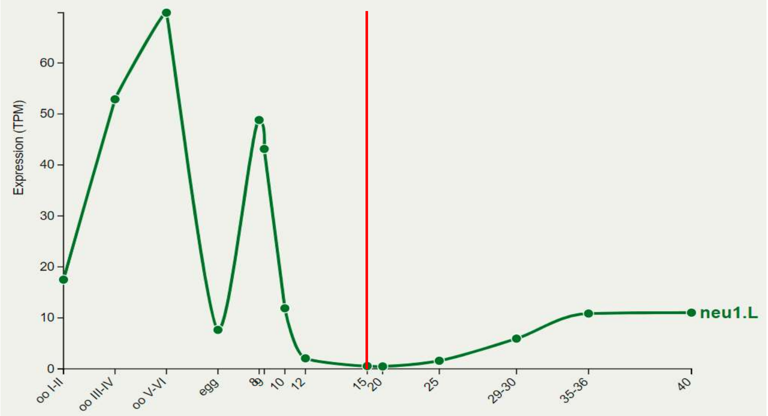
cdc6.L	2,00	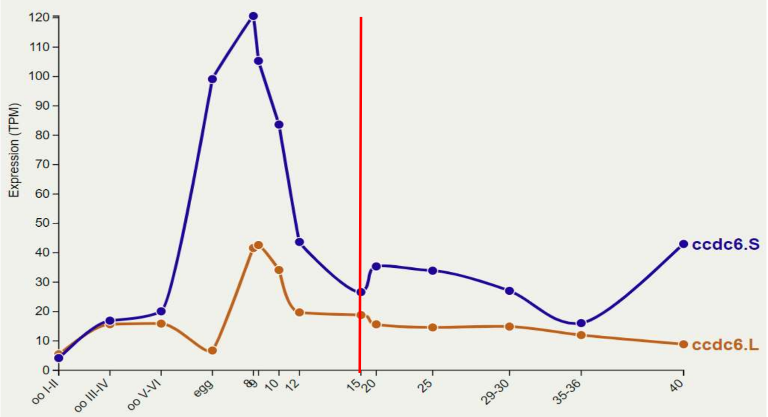
ccna1.L	2,00	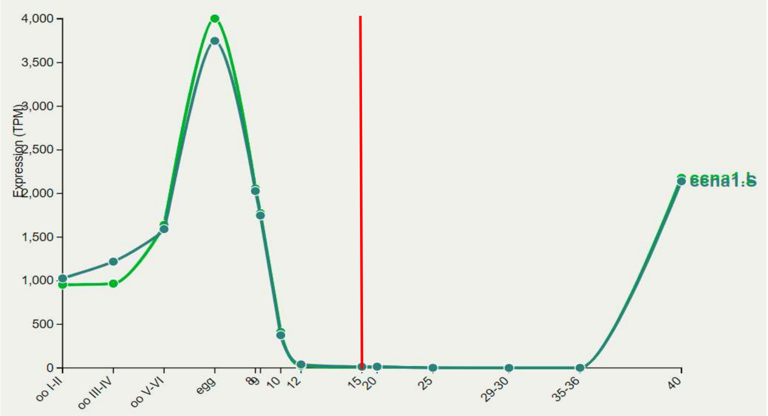

<table>
<tr><th colspan="3">Genes downregulated in embryos injected with insrr MO1 at the middle neurula stage (marked by vertical red line)</th></tr>
<tr><th>Gene</th><th>Expression fold change: insrr MO1/ control MO injected</th><th>Temporal expression pattern</th></tr>
<tr><td>mylfp.L</td><td>0,15</td><td>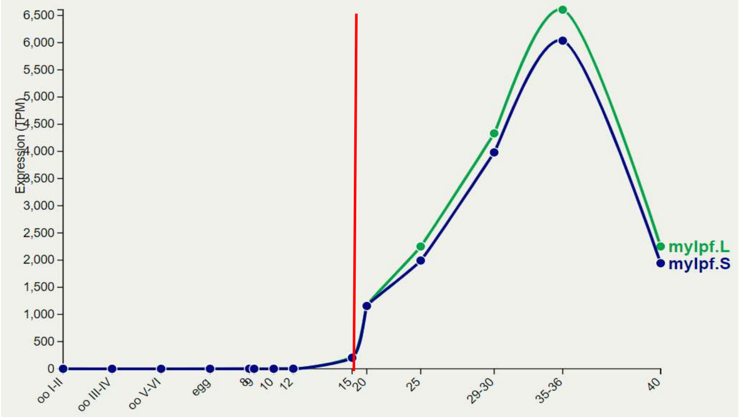
</td></tr>
<tr><td>act3.L</td><td>0,15</td><td>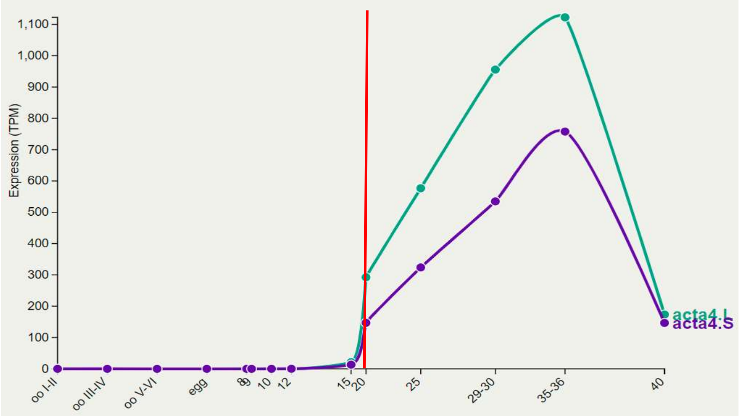
</td></tr>
<tr><td>act2.L</td><td>0,15</td><td>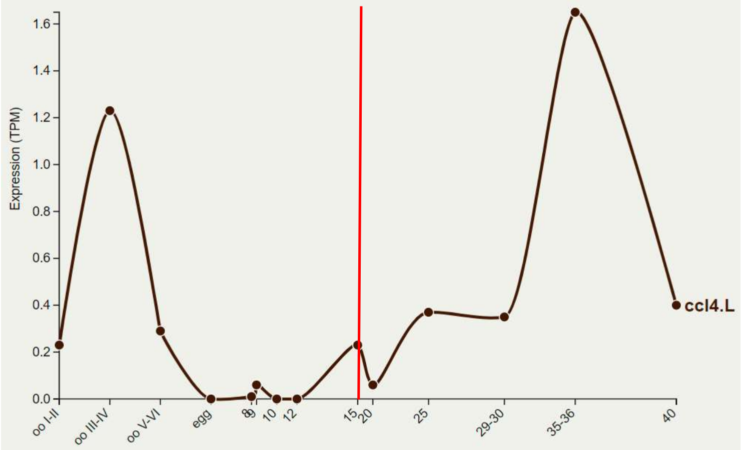
</td></tr>
</table>

myl1.S	0,25	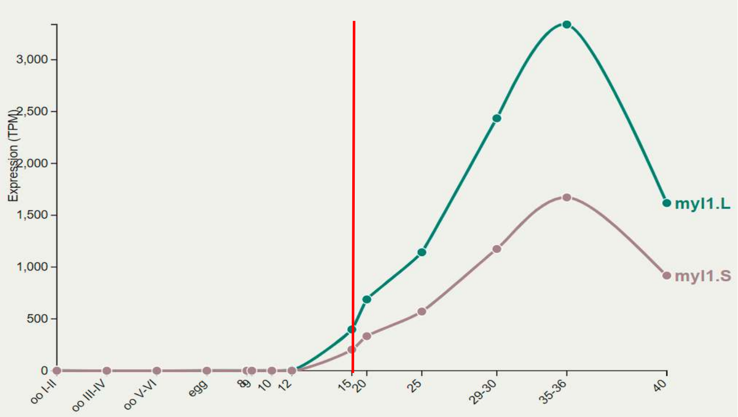
col2a1.L	0,26	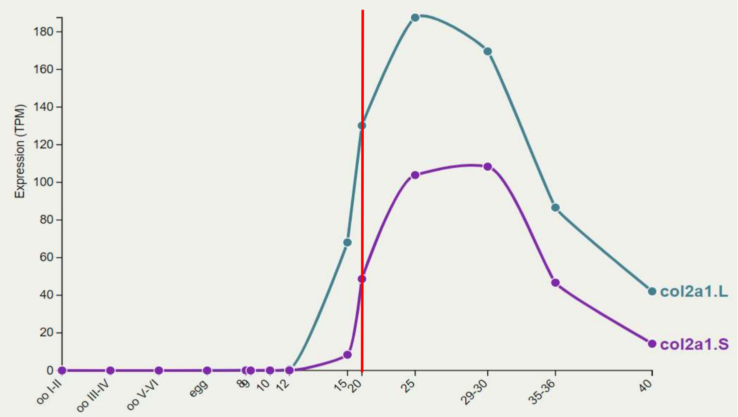
des.1.L	0,26	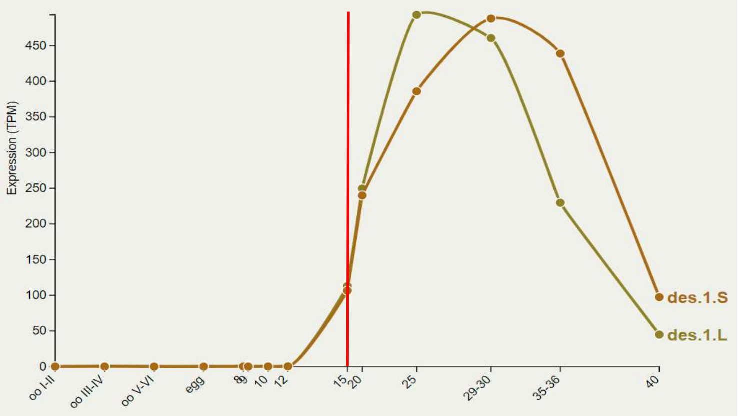
ankrd37.L	0,29	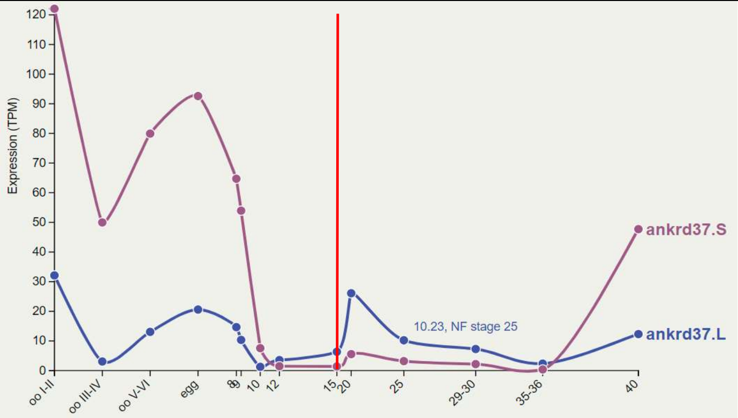

fbxl22.S	0,30	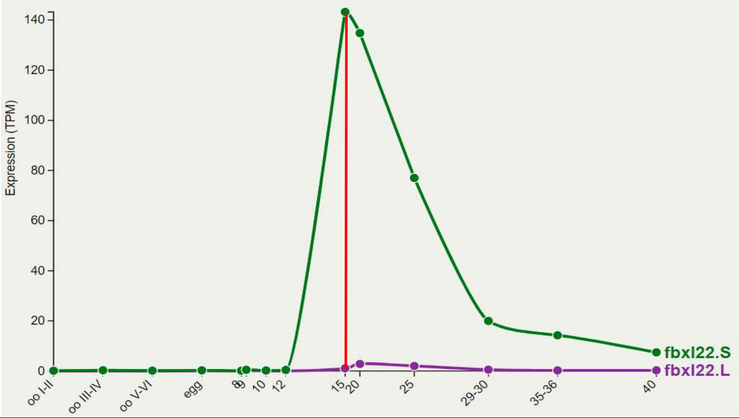

tnnc2.L	0,30	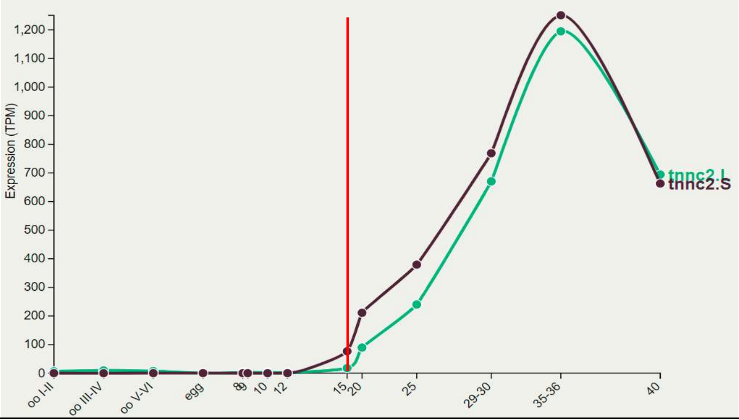
pax6.S	0,30	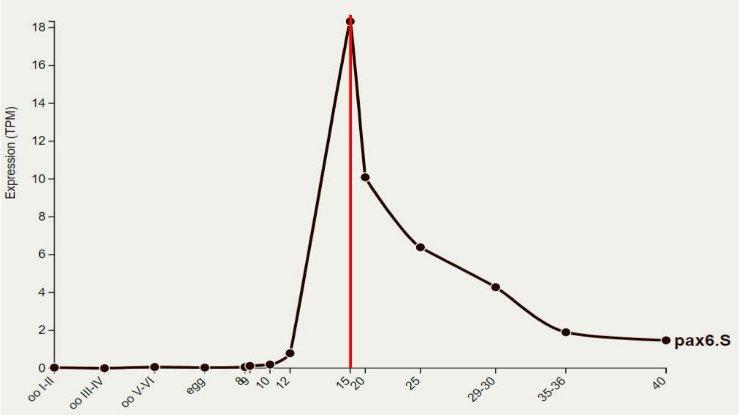
des.1.S	0,30	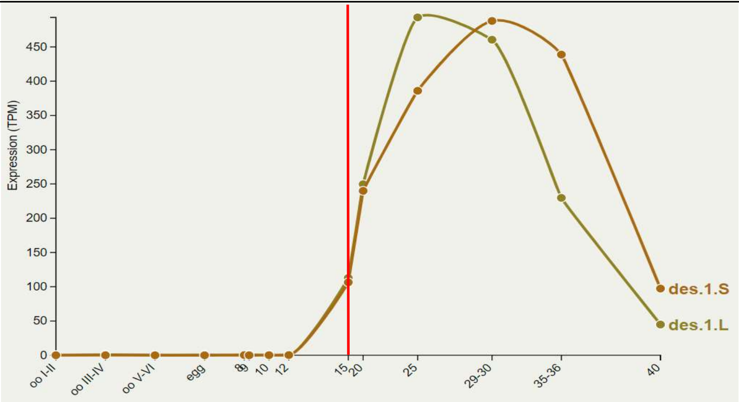

smyd1.L	0,31	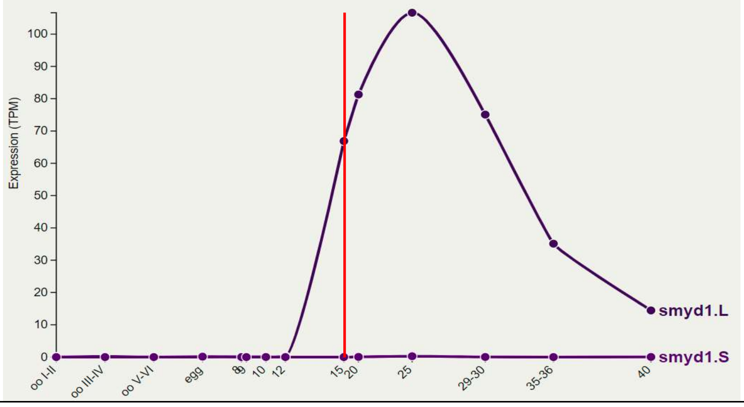

tnnt3.L	0,32	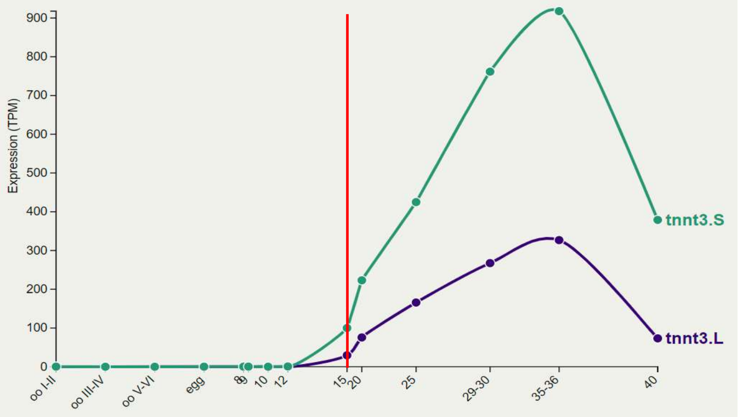
nr2f5.S	0,32	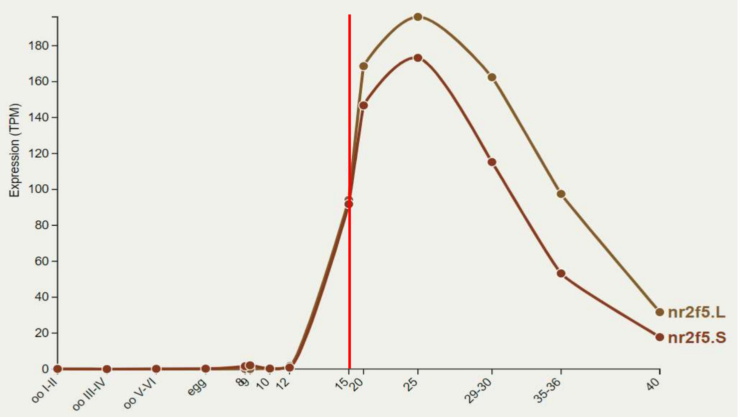
thbs4.S	0,32	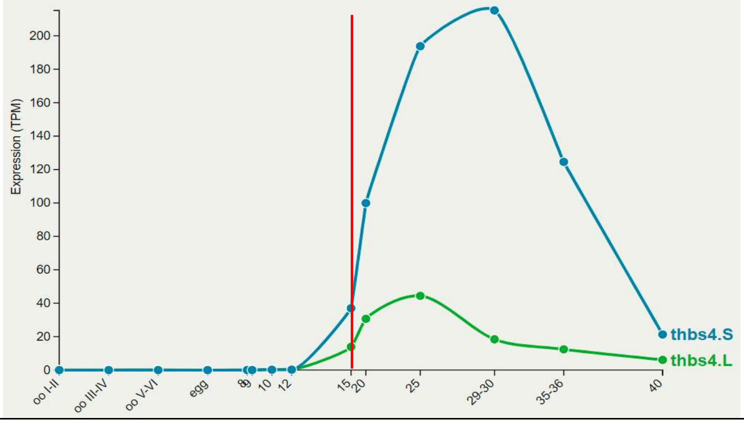
